# Supplementary material for: Resolving the full spectrum of human genome variation using Linked-Reads
Source: Genome Res. 2019 Apr;29(4):635–45. doi: 10.1101/gr.234443.118 (PMC6442396; doi:10.1101/gr.234443.118)
Supplement: Supplemental Material [file supp_gr.234443.118_Supplemental_longranger-2.2.zip › longranger-2.2.tar.bz2!/lr2.2-public/lib/stan/cmdstan/stan/doc/api/html/math_2functions_2multiply__log_8hpp_source.html]

Stan: src/stan/math/functions/multiply\_log.hpp Source File


|  |  |
| --- | --- |
|  | Stan  2.5.0  probability, sampling & optimization |


- Main Page
- Related Pages
- Namespaces
- Classes
- Files

- File List
- File Members

All Classes Namespaces Files Functions Variables Typedefs Enumerations Enumerator Friends Macros Pages

- src
- stan
- math
- functions

multiply\_log.hpp

Go to the documentation of this file.

1 #ifndef STAN\_\_MATH\_\_FUNCTIONS\_\_MULTIPLY\_LOG\_HPP

2 #define STAN\_\_MATH\_\_FUNCTIONS\_\_MULTIPLY\_LOG\_HPP

3

4 #include <boost/math/tools/promotion.hpp>

5

6 namespace stan {

7  namespace math {

8

49  template <typename T\_a, typename T\_b>

50  inline typename boost::math::tools::promote\_args<T\_a,T\_b>::type

51  multiply\_log(const T\_a a, const T\_b b) {

52  using std::log;

53  if (b == 0.0 && a == 0.0)

54  return 0.0;

55  return a \* log(b);

56  }

57

58  }

59 }

60

61 #endif

stan::math::multiply\_log

boost::math::tools::promote\_args< T\_a, T\_b >::type multiply\_log(const T\_a a, const T\_b b)

Calculated the value of the first argument times log of the second argument while behaving properly w...

**Definition:** multiply\_log.hpp:51

stan::math::log

Eigen::Matrix< T, Rows, Cols > log(const Eigen::Matrix< T, Rows, Cols > &m)

Return the element-wise logarithm of the matrix or vector.

**Definition:** log.hpp:16

stan::agrad::log

fvar< T > log(const fvar< T > &x)

**Definition:** log.hpp:15


---

[ Stan Home Page ]

*© 2011–2014,
Stan Development Team.*
